# Identification of natural compounds tubercidin and lycorine HCl against small‐cell lung cancer and BCAT1 as a therapeutic target

**DOI:** 10.1111/jcmm.17246

**Published:** 2022-03-22

**Authors:** Jungang Chen, Lindsey Barrett, Zhen Lin, Samantha Kendrick, Shengyu Mu, Lu Dai, Zhiqiang Qin

**Affiliations:** ^1^ Department of Pathology Winthrop P. Rockefeller Cancer Institute University of Arkansas for Medical Sciences Little Rock Arkansas USA; ^2^ Department of Pathology Tulane University Health Sciences Center Tulane Cancer Center New Orleans Louisina USA; ^3^ Department of Biochemistry and Molecular Biology University of Arkansas for Medical Sciences Little Rock Arkansas USA; ^4^ Department of Pharmacology & Toxicology University of Arkansas for Medical Sciences Little Rock Arkansas USA

**Keywords:** BCAT1, drug screening, lung cancer, natural product, SCLC

## Abstract

Although small‐cell lung cancer (SCLC) accounts for a small fraction of lung cancer cases (~15%), the prognosis of patients with SCLC is poor with an average overall survival period of a few months without treatment. Current treatments include standard chemotherapy, which has minimal efficacy and a newly developed immunotherapy that thus far, benefits a limited number of patients. In the current study, we screened a natural product library and identified 5 natural compounds, in particular tubercidin and lycorine HCl, that display prominent anti‐SCLC activities *in vitro* and *in vivo*. Subsequent RNA‐sequencing and functional validation assays revealed the anti‐SCLC mechanisms of these new compounds, and further identified new cellular factors such as BCAT1 as a potential therapeutic target with clinical implication in SCLC patients. Taken together, our study provides promising new directions for fighting this aggressive lung cancer.

## 1 INTRODUCTION

Small‐cell lung cancer (SCLC) is an aggressive neuroendocrine carcinoma that represents approximately 15% of all lung cancer cases.^1^ Numerous data indicate that SCLC is strongly associated with tobacco use.^2^ Currently, the standard treatments for patients with early‐stage or locally advanced disease include radiation and platinum‐based chemotherapy.^1^ However, most patients have distant metastatic disease at initial diagnosis and require systemic chemotherapy with or without a recently developed immunotherapy. Tumours are initially responsive to these therapies, but the responses are transient in most SCLC patients resulting in a median <2 year survival period for patients with early‐onset disease while only ~1 year for patients with metastatic disease. Although there have been many recent discoveries on the genetic and biological pathways driving SCLC, the current treatments for this disease do not significantly improve the prognosis of the patients, thus still emphasizing the need to develop new and effective treatment.

Recent studies have shown that many natural products from plant or other resources display anti‐cancer activities, or are able to enhance the efficacy of chemotherapy and/or other treatments while not affecting normal cell viability.^3^, ^4^, ^5^ In contrast to these other cancers, there are few natural products which have been reported with anti‐SCLC activities, most likely due to the lack of including SCLC cells in the high‐throughput screening. One recent study reported 3‐bromoascochlorin (BAS), a marine natural product isolated from the coral‐derived fungus *Acremonium sclerotigenum*, GXIMD02501 displays activity against SCLC cell proliferation.^6^ In the current study, we first screened a natural product library and identified hit compounds with selective and prominent anti‐SCLC activities *in vitro* and *in vivo*. RNA‐sequencing analyses revealed the anti‐SCLC mechanisms of these natural compounds and further identified a list of new cellular factors (especially BCAT1) required for SCLC cell survival with clinical implication in SCLC patients. Our results provide new insights into the mechanisms of SCLC pathogenesis and offer promising therapeutic directions for this aggressive lung cancer.

## 2 MATERIALS AND METHODS

### 2.1 Cell culture and reagents

SCLC cell lines, DMS 53 and DMS 114, as well as primary bronchial/tracheal epithelial cells (PBTEC) were all purchased from the American Type Culture Collection (ATCC) and cultured as recommended by the manufacturer. All experiments were carried out using cells harvested at low (<20) passages. A compound library consisting of 756 natural products was purchased from Selleck Chemicals. The SCLC formalin‐fixed, paraffin‐embedded (FFPE) tissue arrays, which contained 80 cases (Cat. #LC818c), and normal lung tissue arrays, which contained 24 cases (Cat. #LCN241), were purchased from US Biomax.

### 2.2 *High*‐*throughput screening*

SCLC cell line DMS 114 was seeded into 96‐well plates for 24 h; then, the natural product compounds were added into the wells at a final concentration of 10 µM for an additional 72‐h treatment. The cytotoxicity against SCLC was measured using the WST‐1 cell proliferation assays (Roche). Briefly, after the period of treatment of cells, 10 μL/well of cell proliferation reagent, WST‐1 (4‐[3‐(4‐Iodophenyl)‐2–^4^‐2H‐5‐tetrazolio]‐1,3‐benzene disulfonate), was added and incubated for 3 h at 37°C in 5% CO_2_. The absorbance of samples was measured by using a microplate reader at 490 nm. Data were normalized as the inhibition ratio to the DMSO control.

### 2.3 Cell apoptosis assays

Flow cytometry was used for the quantitative assessment of apoptosis with the FITC‐Annexin V/propidium iodide (PI) Apoptosis Detection Kit I (BD Pharmingen) on a FACS Calibur 4‐colour flow cytometer (BD Bioscience).

### 2.4 Colony formation assays

The tumour cell anchorage‐independent growth abilities were assessed using the Elplasia round‐bottom plates (Corning) according to the manufacturer's protocol with brief modifications as follows. In 96‐well plates, a volume of 50 μL containing cell culture medium with the tested drugs or DMSO vehicle per well was added to pre‐wet the wells, and then, the plate was centrifuged at 500 *g* for 1 min. Next, the same volume of cell suspension (~5 × 10^4^ cells/ml) was added into the wells and cultured in an incubator at 37°C for about 1 week. The number of colonies in each well was recorded and calculated by using the Olympus IX83 microscope.

### 2.5 SCLC xenograft models

Cells were counted and washed once in ice‐cold sterile PBS; then, 6 × 10^5^ DMS 114 cells in 50 µL PBS plus 50 µL growth factor‐depleted Matrigel (BD Biosciences) were injected subcutaneously into the flank of nude mice, 6‐ to 8‐week‐old, male/female (Jackson Laboratory). When tumours reached 8–10 mm in diameter (~1.5 weeks), the mice were randomly separated into different groups (4 mice per group) and received *in situ* subcutaneous injection with either vehicle, tubercidin (5 mg/kg) or lycorine HCl (10 mg/kg), 3 days/week. The mice were observed and measured every 2–3 days for the size of palpable tumours for an additional 3 weeks. At the end of experiment, the tumours were excised and compared. All the animal protocols were approved by the UAMS Animal Care and Use Committees in accordance with national guidelines.

### 2.6 *RNA*‐*sequencing and enrichment analysis*

RNA‐sequencing of triplicate samples was performed by BGI Americas Corporation using their unique DNBSEQ™ sequencing technology. The completed RNA‐sequencing data were submitted to NCBI Sequence Read Archive (SRA# PRJNA770672). Raw sequencing reads were analysed using the RSEM software (version 1.3.0; human GRCh38 genome sequence and annotation), and gene expression was quantified as previously described.^7^ The EBSeq software was utilized to call differentially expressed genes that were statistically significant using a false discovery rate (FDR) less than 0.05. Differentially expressed genes between natural compounds‐ and vehicle‐treated SCLC cells were used as input for the GO_enrichment analyses.

### 2.7 RNA interference (RNAi)

For RNAi assays, BCAT1 On‐Target plus SMARTpool small interfering RNA (siRNA; Dharmacon) or negative control siRNA were delivered using the DharmaFECT transfection reagent as recommended by the manufacturer.

### 2.8 Western blot

Total cell lysates (20 µg) were resolved by 10% SDS‐PAGE, transferred to nitrocellulose membranes and immunoblotted with antibodies to BCAT1 (Abcam), Ras, phosphor (p)‐BRaf (Ser445)/total (t)‐BRaf, p‐MEK (Ser217/221)/t‐MEK, p‐ERK (Thr202/Tyr204)/t‐ERK and p‐Akt (Ser473)/t‐Akt (Cell Signaling). β‐Actin served as the loading control (Cell Signaling). Immunoreactive bands were identified using an enhanced chemiluminescence reaction (Perkin‐Elmer) and visualized by autoradiography.

### 2.9 Immunohistochemistry

Immunohistochemistry was performed using the Avidin‐Biotin‐Peroxidase complex, according to the manufacturer's instructions (Vector Laboratories) as described previously.^8^, ^9^ The rabbit polyclonal anti‐BCAT1 (Abcam) was used at 1:50 dilution. Tissue array slides were then scanned with an Aperio CS2 digital pathology scanner. Images were obtained with Aperio ImageScope software (Leica) at 40× magnification. The percentage of DAB stained pixels were determined by analysing the raw images with the QuPath software (version 0.2.3).^10^

### 2.10 Statistical analysis

Significant differences between experimental and control groups were determined using the two‐tailed Student's *t*‐test. The 50% cytotoxicity concentrations (CC_50_) were calculated by using SPSS v20.0.

## 3 RESULTS

### 3.1 High‐throughput screening and identification of new natural compounds displaying anti‐SCLC activities

**FIGURE 1:**
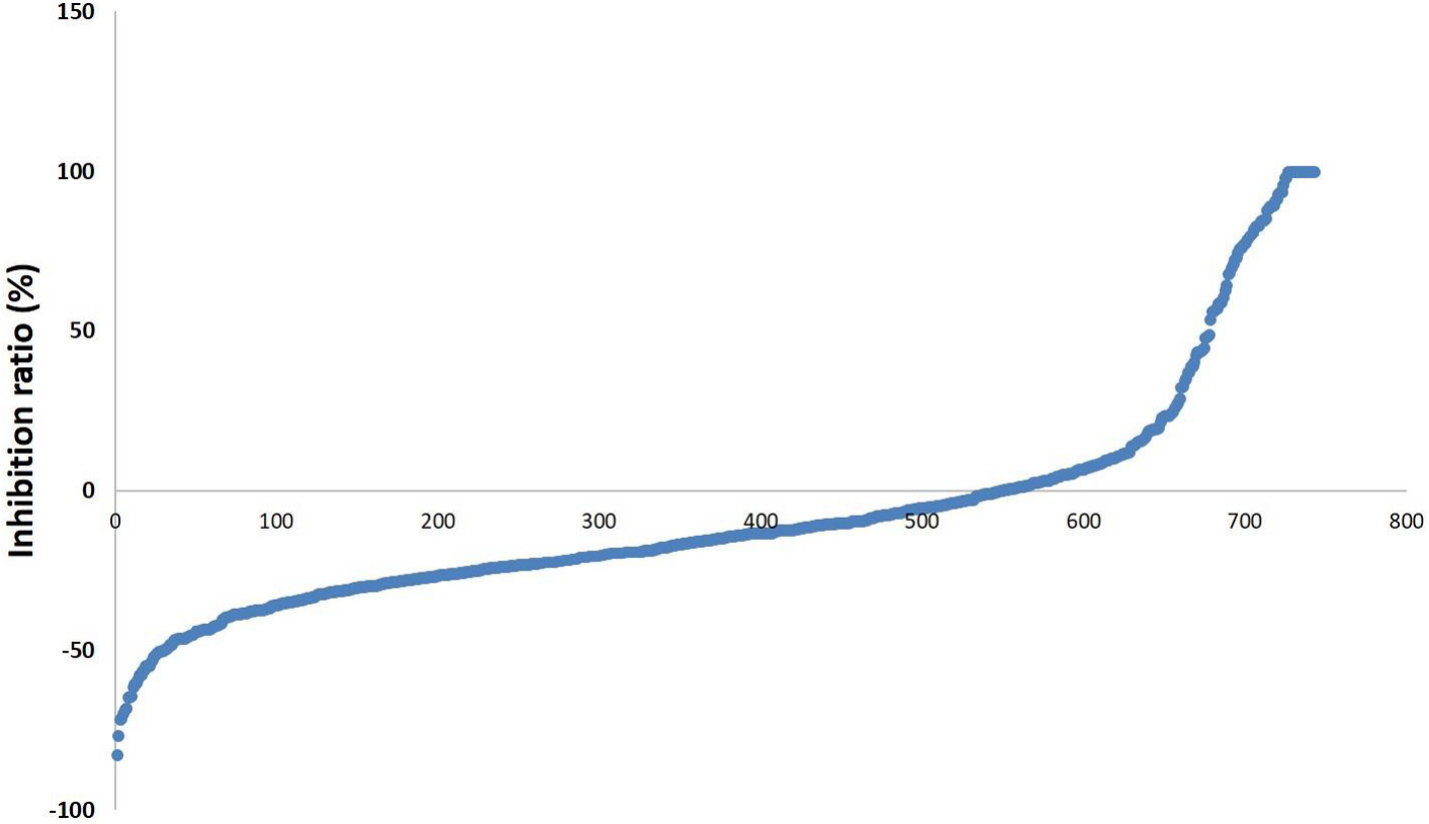
High‐throughput screening and identification of new natural compounds with anti‐SCLC activities. Primary screening results of 756 natural compounds against SCLC, which are arranged in order of inhibition rate. The natural compounds in source plates were delivered at 10 µM (final concentration) to 96‐well plates seeded with SCLC cell line DMS 114 for 72 h treatment; then, cell proliferation was examined using the WST‐1 cell proliferation assays (Roche)

**FIGURE 2:**
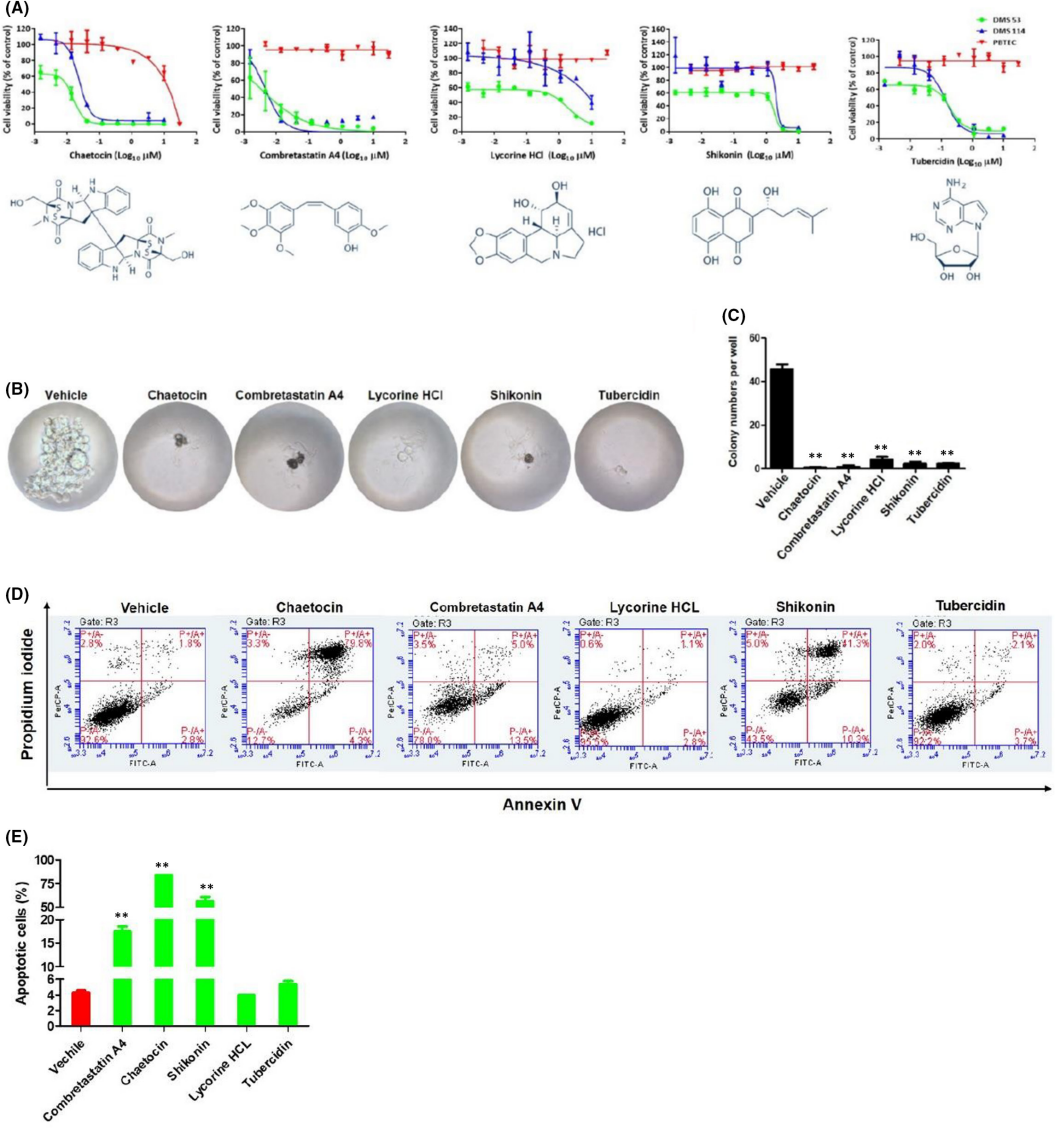
Hit natural compounds prominently inhibit SCLC cell growth. (A) The structures of the final 6 hit compounds and their dose‐dependent ‘killing curves’ on SCLC cells. (B, C) The inhibition of DIPG anchorage‐independent growth ability by new natural compounds, using colony formation assays. (D, E) DMS 114 cells were treated with natural compounds or vehicle for 48 h; then, cell apoptosis was measured by Annexin V‐PI staining and flow cytometry analysis. Error bars represent S.D. for 3 independent experiments, **p < 0.01 (vs the vehicle control)

One of commercial SCLC cell lines, DMS 114, derived from a surgical biopsy of a SCLC patient, was used for our screening assays. After screening a chemical library containing 756 natural products, we found 94 hit compounds induced prominent cytotoxicity (>50%) at 10 µM concentration (Figure 1). After searching published literature, we then excluded molecules with known anti‐cancer activities (e.g. paclitaxel, docetaxel, doxorubicin and rapamycin), which yielded 76 novel compounds. After calculating the CC_50_ using drug‐killing curves on both DMS 52 and DMS 114 cell lines, we ultimately identified 5 hit compounds with 50% cytotoxicity concentrations (CC_50_) <10 µM, most of them effective at nM levels (Table 1 and Figure 2A). These remaining compounds were Chaetocin (a histone methyltransferase inhibitor), Combretastatin A4 (a microtubule‐targeting agent), lycorine HCl (a putative inhibitor of focal adhesion kinase pathway), Shikonin (a putative cell cycle and apoptosis inducer) and tubercidin (an adenosine analogue). Notably, all of the final hit compounds showed little or no cytotoxicity on normal primary bronchial/tracheal epithelial cells (PBTECs), making them highly selective towards SCLC cells and suitable for drug development with a selective index (SI) of ~15–8000 (Table 1).

**TABLE 1:** Prominent anti‐SCLC activities of hit natural products

Compounds	CAS	CC_50_ (µM)^a^			SI^c^
		DMS 53	DMS 114	PBTEC^b^	
Chaetocin	28097‐03‐2	0.016	0.024	~20	~1000
Combretastatin A4	117048‐59‐6	0.0033	0.0045	>30	>7692.31
Lycorine HCl	2188‐68‐3	1.9	0.68	>30	>23.26
Shikonin	54952‐43‐1	1.75	2.04	>30	>15.83
Tubercidin	69‐33‐0	0.19	0.14	>30	>181.82

^a^ CC_50_: the 50% cytotoxic concentration determined by using the WST‐1 assay.

^b^ PBTEC: primary bronchial/tracheal epithelial cells.

^c^ SI (Selective Index): CC_50_ of PBTEC / CC_50_ of SCLCs.

Next, using the colony formation assays, we observed all 5 hit compounds dramatically inhibited anchorage‐independent growth of SCLC cells when compared to the vehicle control. The vehicle‐treated SCLC cells tended to form numerous, large colonies, in contrast, treatment with the hit compounds significantly reduced the number of colony formation with observable cell debris in the wells (Figure 2B,C). Using FITC‐Annexin V/propidium iodide (PI) staining combined with flow cytometry analysis, we found that 3 of the 5 hit compounds (Chaetocin, Combretastatin A4 and Shikonin) significantly induced SCLC cell apoptosis, including the increased subpopulation of both early (Annexin V+/PI−) and late (Annexin V+/PI+) apoptotic cells, when compared to the vehicle‐treated cells (Figure 2D,E).

### 3.2 The new natural compounds effectively repress SCLC cell growth in vivo

**FIGURE 3:**
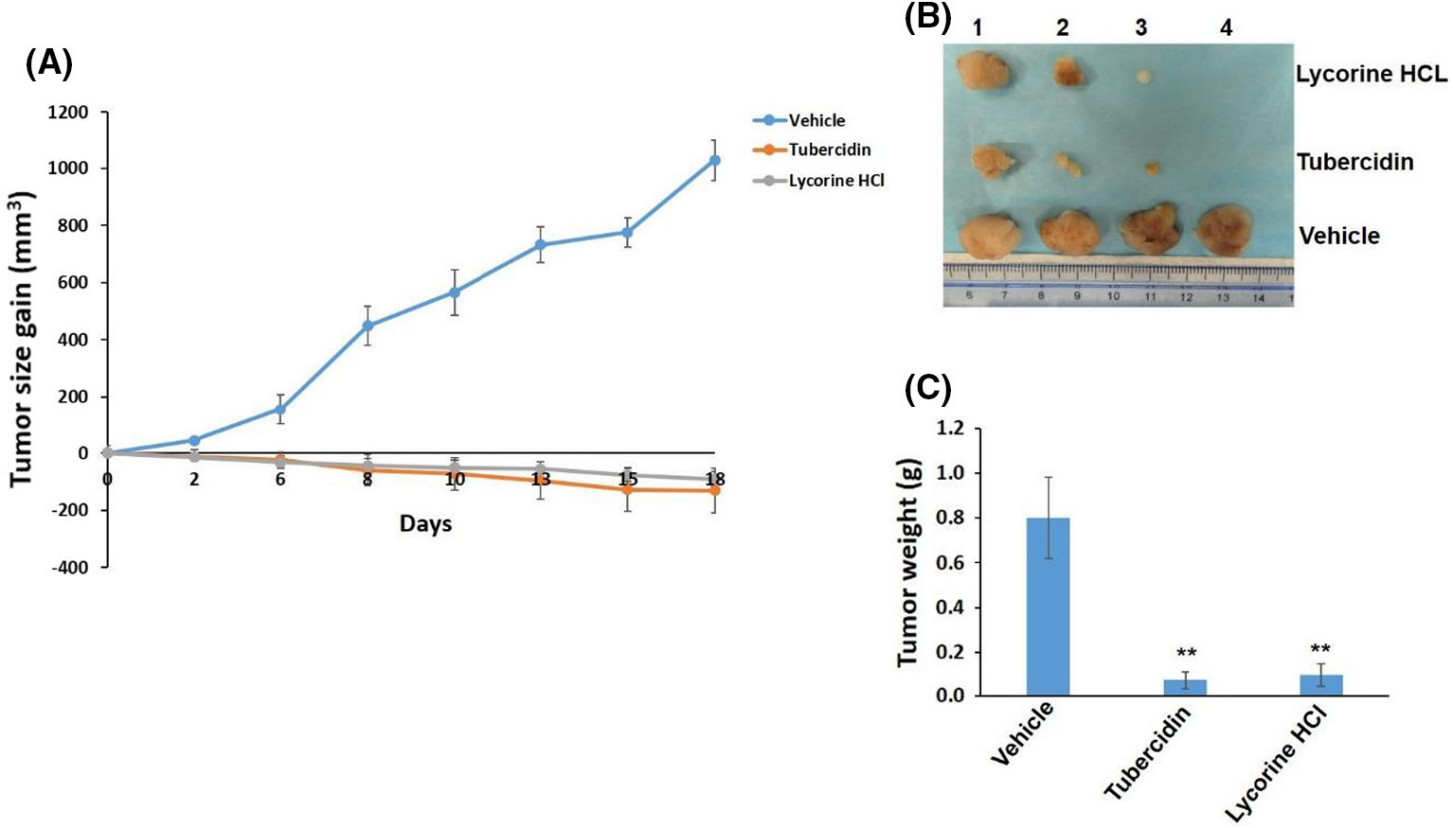
New natural compounds displaying effective anti‐SCLC activities *in vivo*. (A) 6 × 10^5^ DMS 114 cells in 50 µL PBS plus 50 µL growth factor‐depleted Matrigel were injected subcutaneously into the flank of nude mice. When tumours reached 8–10 mm in diameter (~1.5 weeks), the mice were randomly separated into different groups (4 mice per group) and received *in situ* subcutaneous injection with either vehicle, tubercidin (5 mg/kg) or lycorine HCl (10 mg/kg), 3 days/week. The mice were observed and measured every 2–3 days for the size of palpable tumours for additional 3 weeks. (B, C) At the end of treatments, the tumours were excised for weighing and size comparison, **p < 0.01 (vs the Vehicle control)

To assess *in vivo* efficacy of our new natural compounds, we tested tubercidin and lycorine HCl in an established SCLC xenograft mice model. Our results indicated that both tubercidin and lycorine HCl treatments significantly repressed DMS 114 tumour growth in mice (Figure 3A). At the end of treatments, the tumours were excised for size and weight comparison. We found that the mice from both tubercidin and lycorine HCl‐treated groups formed much smaller tumours when compared to the vehicle‐treated group. Notably, the tumours from one mice in both tubercidin and lycorine HCl‐treated groups exhibited a complete response and were no longer detectable at the end of treatment (Figure 3B,C). Together, these data demonstrate significant *in vivo* efficacy of the two new natural compounds against SCLC, positing them as promising therapeutic agents.

### 3.3 Transcriptomic analysis of gene profiling in SCLC cells altered by natural compounds

**FIGURE 4:**
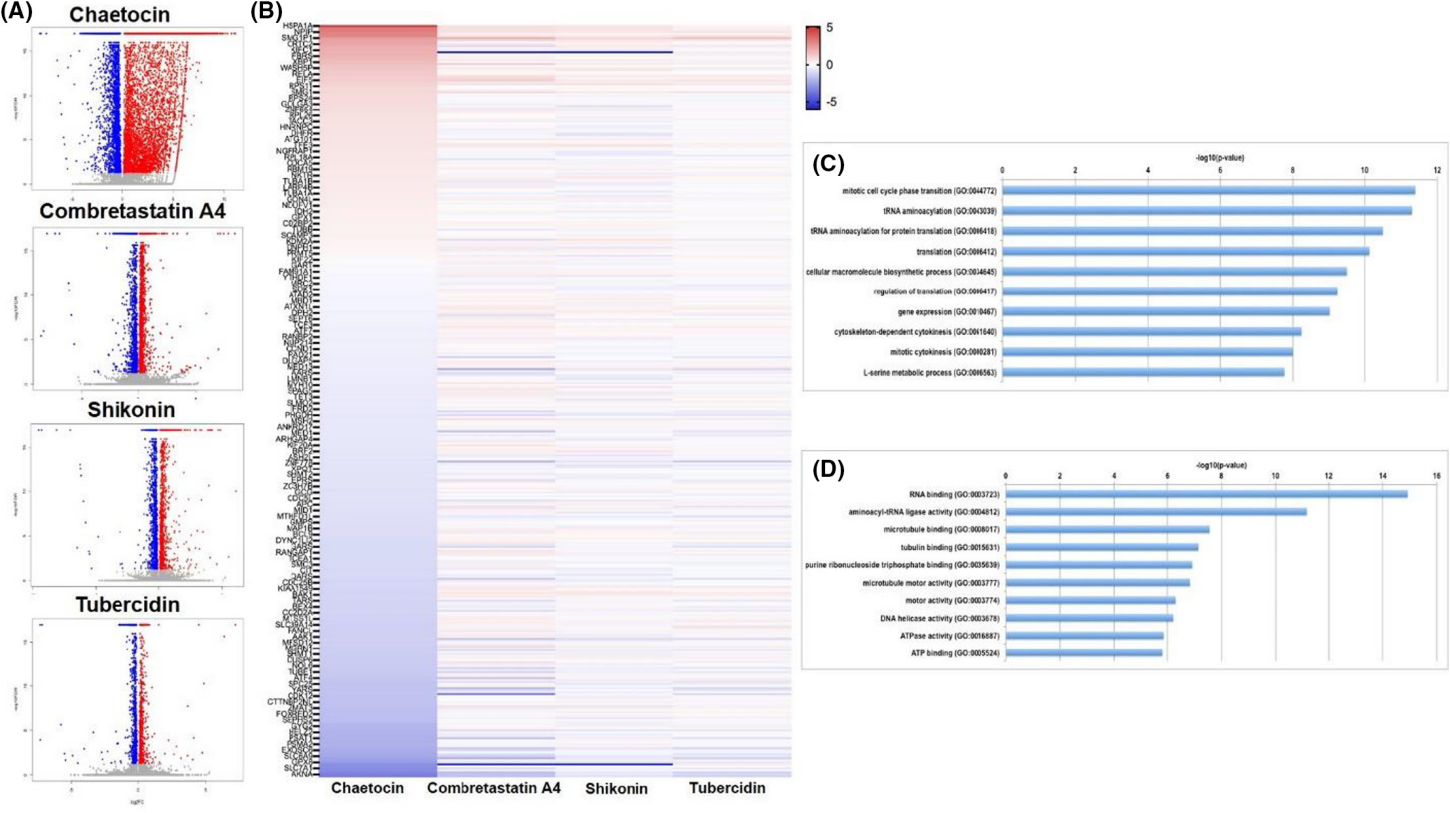
Transcriptome analysis of new natural compound‐treated SCLC cells. (A) RNA‐sequencing was used to investigate changes in the transcriptome between natural compounds and vehicle‐treated DMS 114 SCLC cells. The significantly altered genes (*p* < 0.05) are shown in the volcano plot panels. (B) The heat map of commonly altered genes expression by 4 hit natural compounds in SCLC. (C, D) The GO_enrichment (biological process and molecular function) analysis of the commonly changed cellular genes by 4 hit natural compounds in SCLC

To determine the global cellular changes induced by these hit compounds, we compared the gene profiles of vehicle‐ to compound‐treated tumour cells (Chaetocin, Combretastatin A4, Shikonin and tubercidin) by using RNA‐sequencing analyses. The volcano plots showed the scattering of genes which were significantly up‐regulated or down‐regulated (FDR <0.05) in these hit compound‐treated DMS 114 cells (Figure 4A). Combretastatin A4, Shikonin and tubercidin caused fewer changes in cellular gene expression in SCLC cells relative to the significant changes induced by Chaetocin. This differential impact on the transcriptome profile was confirmed by intersection analysis shown in a heat map (Figure 4B). The heat map indicated that there were ~300 transcripts, including some non‐coding RNAs, which were commonly and significantly altered in all of 4 natural compound‐treated SCLC cells. Interestingly, we discovered even if the same genes were targeted by more than one compound in the SCLC‐treated cells, the gene may display a different trend in expression (up‐ or down‐regulation) in response to different compounds, indicating these compounds may use varied mechanisms or targets to cause SCLC cell death. However, the GO_enrichment analysis of these common gene‐target candidates altered by the four compounds identified several major functional categories are potentially involved. The biological process module analysis indicated many of the genes belong to pathways important for mitotic cell cycle phase transition, tRNA aminoacylation, cellular macromolecule biosynthetic process and cytoskeleton‐dependent cytokinesis (Figure 4C). In support, the molecular function module analysis showed the majority of the genes associate with similar processes such as RNA binding, aminoacyl‐tRNA ligase activity, microtubule/tubulin binding and purine ribonucleoside triphosphate binding (Figure 4D).

### 3.4 Identification of BCAT1 as a new cellular gene required for SCLC cell survival

**FIGURE 5:**
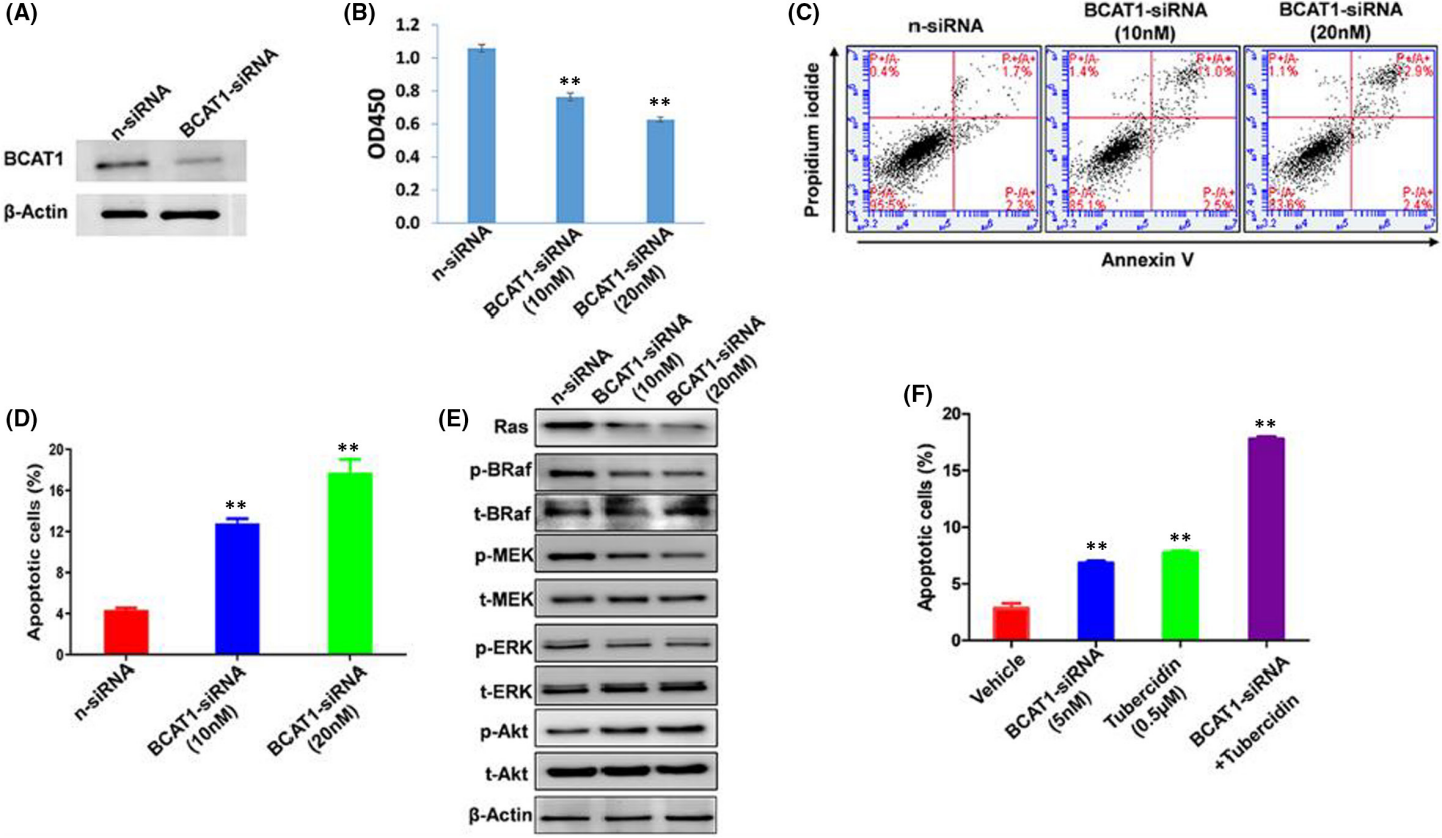
BCAT1 is required for SCLC cell survival. (A–E) DMS 114 cells were transfected with BCAT1‐siRNA or non‐target control siRNA (n‐siRNA) for 72 h; then, protein expression, cell proliferation and apoptosis were measured by using Western blot, WST‐1 assays and flow cytometry analysis respectively. (F) Cells were transfected with BCAT1‐siRNA with or without tubercidin treatment for 48 h; then, cell apoptosis was measured as above. Error bars represent S.D. for 3 independent experiments, **p < 0.01 (vs the n‐siRNA control or the vehicle control)

We propose that certain gene candidates identified from RNA‐sequencing analysis are potentially related to SCLC survival and/or pathogenesis. Thus, we selected BCAT1 (branched‐chain amino acid transaminase 1) for subsequent functional validation, since its expression was dramatically down‐regulated in SCLC cells following treatment of all 4 natural compounds. BCAT1 is the predominant isoform of BCAT that initiates the catabolism of branched‐chain amino acids (BCAAs).^11^ Recent data report that BCAT1 expression is highly expressed in multiple cancers and required for individual cancer progression.^12^, ^13^, ^14^ Consistent with this previous work, we demonstrated successful knockdown of BCAT1 by RNAi effectively repressed SCLC cell proliferation through inducing tumour cell apoptosis (Figure 5A–D). To determine a potential mechanism for loss of BCAT1‐induced cell death, we screened several signalling pathways related to cell proliferation. We found knockdown of BCAT1 significantly reduced the activities of Ras/BRaf/MEK/ERK signalling kinases in SCLC cells (Figure 5E), indicating this pathway is involved in BCAT1‐mediated cancer cell proliferation. We also observed a synergistic effect on inducing SCLC cell apoptosis with a combination of knockdown of BCAT1 and tubercidin treatment (Figure 5F). Taken together, our data confirmed an important role of BCAT1 in SCLC cell survival and proliferation, which may represent an attractive therapeutic target for SCLC.

### 3.5 Clinical implications of BCAT1 in SCLC patients

**FIGURE 6:**
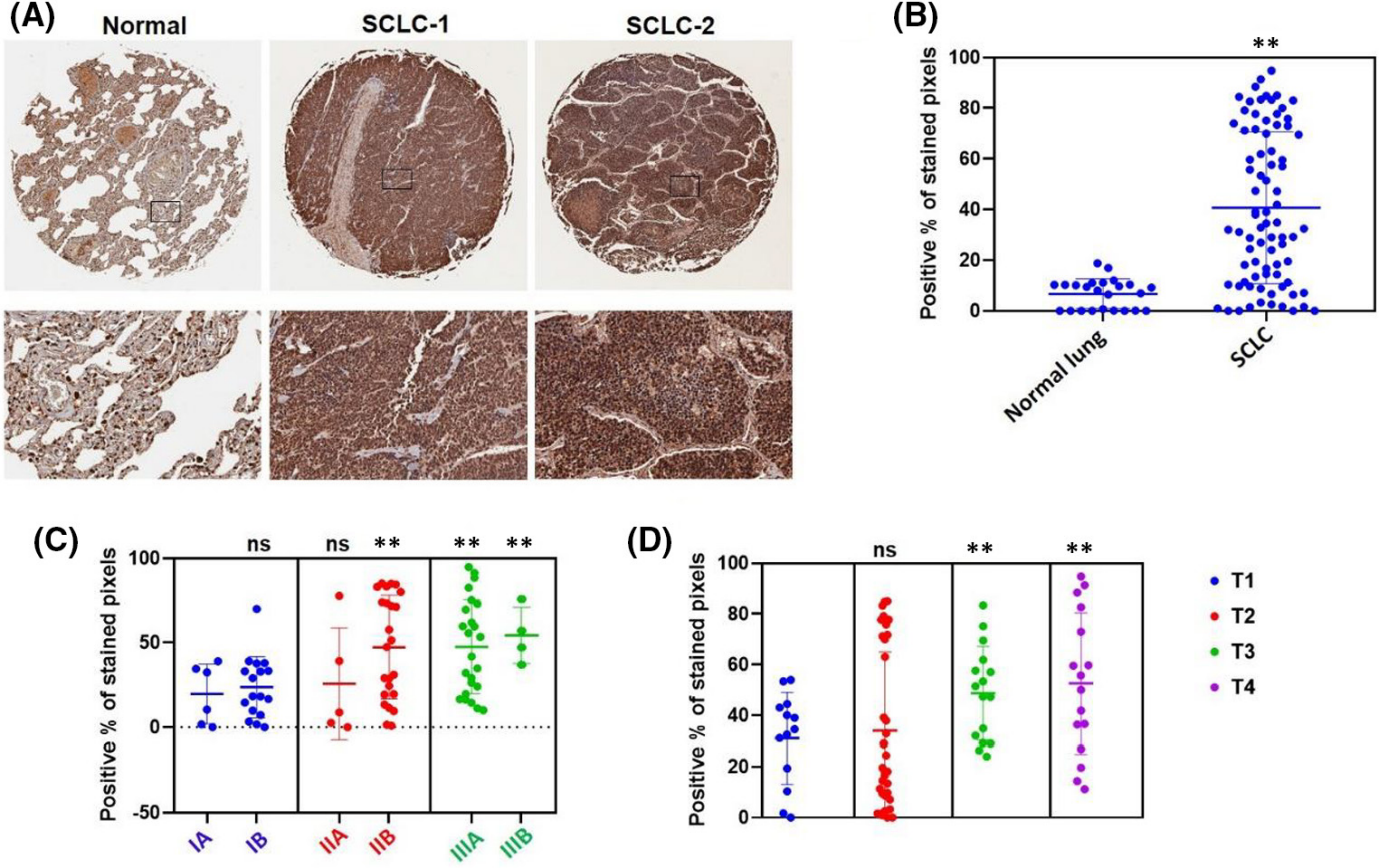
Clinical relevance of BCAT1 in SCLC. Expression of EIF4G1 in formalin‐fixed paraffin‐embedded (FFPE) SCLC and normal lung tissue arrays were determined using immunohistochemistry (IHC). (A) The IHC images from representative cases. (B–D) The percentage of DAB stained pixels were determined by analysing the raw images with the QuPath software (version 0.2.3). The nested graphs show expressional difference among these groups. **p < 0.01 (vs the normal lung tissue group, the IA group or the T1 group respectively); ns: not significant

SCLC tissue arrays containing 80 cases together with normal lung tissue arrays composed of 24 cases, were used to explore the clinicopathological role of BCAT1 in SCLC progression through immunohistochemistry (IHC) staining. The IHC results indicated that the expression levels of BCAT1 protein were significantly up‐regulated in SCLC tumour tissues compared to normal lung tissues (Figure 6A,B), although its expression was variable among tumour tissues from different SCLC patients. Based on clinical characteristics of the patient cases, we observed the expression levels of BCAT1 were significantly increased in advanced stages of SCLC, especially Stages IIB, IIIA and IIIB (Figure 6C). Furthermore, based on TNM scores, the expression of BCAT1 was significantly higher in T3 and T4 (Figure 6D). Taken together, these clinical data strongly support a role of BCAT1 in SCLC development and progression.

## 4 DISCUSSION

Although SCLC accounts for a small portion of lung cancer cases, SCLC patients present with a worse prognosis than non‐small‐cell lung cancer (NSCLC), the major histological type of lung cancer.^15^, ^16^ The advent of recently developed immune checkpoint inhibitors (ICIs) has resulted in significant survival benefits in NSCLC patients. In contrast, clinical trials testing ICIs in SCLC patients have thus far yielded modest benefits, and only a small subset of patients achieve long‐term benefit.^17^ Therefore, new treatments against SCLC are still an urgent clinical need. In the current study, we identified new natural compounds with prominent anti‐SCLC activities (while showing almost no cytotoxicity on normal lung epithelial cells), using high‐throughput screening methods. Notably, all of the final hit compounds have no known association with SCLC treatment, although previous studies show anti‐cancer activity in NSCLC or other cancers. For example, Chaetocin, a fungal metabolite, has been shown to suppress NSCLC cell growth through inducing endoplasmic reticulum (ER) stress and cell apoptosis mediated by death receptor 5 (DR5).^18^ Interestingly, Chaetocin has also been found to induce glioma cell apoptosis in a reactive oxygen species (ROS)‐dependent manner, which potentially occur through the ATM‐YAP1 signalling axis and JNK‐dependent inhibition of glucose metabolism.^19^ Another hit compound, Shikonin, a natural naphthoquinone compound has been shown to inhibit a wide range of human cancer cell lines growth, mainly through inducing a robust up‐regulation of p21 expression and eventual apoptotic cell death.^20^ These observations are consistent with our findings in this current study, as both Chaetocin and Shikonin induced strong apoptosis of SCLC cells. However, we also noticed apoptosis did not occur in SCLC cell response to treatment with lycorine HCL and tubercidin, which requires further investigation.

Although our next‐generation sequencing (NGS) analysis indicated that the hit compounds potentially utilize different mechanisms to kill NSCLC cells, we still see overlapping functional categories affected by these compounds, which supports some common targets in cancer cell survival and proliferation. One interesting category is tRNA aminoacylation, which is performed by aminoacyl‐tRNA synthetases (ARSs). ARSs represent essential and ubiquitous house‐keeping enzymes responsible for charging amino acids to their cognate tRNAs, then providing the substrates for protein synthesis. However, some recent studies revealed a role of multiple ARSs in pathology and their potential use as pharmacological targets and therapeutic agents.^21^ In particular, the up‐regulation of ARSs has been observed in several types of cancer.^22^, ^23^ In addition, the polymorphisms in ARSs genes have been reported to be associated with breast cancer risk^24^; however, the roles of ARSs in SCLC pathogenesis remain largely unknown. Interestingly, the expression of a specific ARSs, methionyl‐tRNA synthetase (MRS), has been found elevated in NSCLC tissues, and its overexpression is associated with poor clinical outcomes in NSCLC patients.^25^ Recently, MRS is further reported to serve as a useful diagnostic marker for lymph node metastasis in NSCLC.^26^ Therefore, we are interested in understanding how these natural product treatments may affect tRNA aminoacylation in SCLC cells.

Here, we selected BCAT1, one of the gene candidates identified by RNA‐sequencing analysis significantly down‐regulated in SCLC cells treated with all 4 hit compounds, for functional validation. We found that knockdown of BCAT1 effectively repressed SCLC cell proliferation potentially through the regulation of Ras/BRaf/MEK/ERK signalling pathway. Interestingly, a study last year reported that knockdown of BCAT1 suppressed melanoma cell proliferation and migration, which was associated with reduced oxidative phosphorylation.^27^ Another recent study found that BCAT1 was able to activate PI3K/Akt/mTOR signalling pathway, contributing to the angiogenesis and tumorigenicity of gastric cancer cells,^28^ although we did not observe repression of Akt phosphorylation by knockdown of BCAT1 in SCLC cells (Figure 5E). Therefore, we propose that BCAT1 may interact and regulate multiple cellular signalling pathways to exert its functions, which are most likely cancer type‐specific. Our tissue‐array analysis further confirmed the clinicopathological role of BCAT1 in SCLC progression. In addition, other studies report high BCAT1 expression was associated with poor patient survival in several types of cancer.^28^, ^29^ One of the remaining questions is how these natural compounds can affect BCAT1 expression. Currently, there are limited data about the regulation of BCAT1 expression, although one study suggests that BCAT1 may be regulated by the transcription factor c‐Myc.^30^

## CONFLICT OF INTEREST

All the authors declare no competing interests.

## AUTHOR CONTRIBUTIONS

Jungang Chen: Data curation (equal); Formal analysis (equal); Investigation (equal). Lindsey Barrett: Formal analysis (equal); Investigation (equal); Writing – original draft (equal). Zhen Lin: Formal analysis (equal). Samantha Kendrick: Formal analysis (equal); Writing – review & editing (equal). Shengyu Mu: Formal analysis (equal); Writing – review & editing (equal). Lu Dai: Conceptualization (equal); Supervision (equal); Writing – review & editing (equal). Zhiqiang Qin: Conceptualization (lead); Supervision (lead).

## AUTHORS’ CONTRIBUTIONS

L. Dai and Z. Qin involved in conception and design, and study supervision. Z. Lin and L. Dai involved in development of methodology. J. Chen, L. Barrett, Z. Lin and L. Dai involved in acquisition of data (provided animals, acquired and managed patients, provided facilities, etc.). J. Chen, L. Barrett, Z. Lin, S. Kendrick, S. Mu and Z. Qin involved in analysis and interpretation of data (e.g. statistical analysis, biostatistics, computational analysis). J. Chen, L. Barrett, S. Kendrick, L. Dai and Z. Qin involved in writing, review and/or revision of the manuscript. Z. Lin and Z. Qin involved in administrative, technical or material support (i.e. reporting or organizing data, constructing databases).

## Data Availability

The data sets used and/or analysed during the current study are available from the corresponding author on reasonable request.
